# Supplementary material for: Are carbon emissions trading and green financial instruments synergistic? -Comprehensive quantitative research based on content analysis
Source: PLoS One. 2024 Mar 7;19(3):e0298601. doi: 10.1371/journal.pone.0298601 (PMC10919601; doi:10.1371/journal.pone.0298601)
Supplement: S1 Data — (ZIP) [file pone.0298601.s001.zip › Data/地级市Z_F工作报告.zip!/║╙─╧/┬σ╤⌠╩╨/└┤╫╘╣1⁄2╓┌║┼í╛┬φ┐╦╩2╛▌═°í┐_2013.docx]

各位代表：

现在，我代表市人民政府向大会作工作报告，请予审议，并请各位政协委员和列席人员提出意见。

一、2012年工作回顾

2012年是我市发展进程中极具挑战的一年。国际金融危机的影响进一步扩大，世界经济持续低迷，国内经济增速放缓。面对复杂严峻的形势，在省委、省政府和市委的正确领导下，在市人大、政协的监督支持下，全市人民紧紧围绕福民强市总体目标，克难攻坚，开拓进取，持续提升 “六加一”攻坚战，经济社会保持了平稳较快发展的态势。预计全年完成地区生产总值3000亿元左右，比上年增长10%；地方公共财政预算收入205亿元左右，增长15%；固定资产投资2280亿元左右，增长23%；社会消费品零售总额1110亿元左右，增长15.4%；外贸出口11.8亿美元左右；城镇居民人均可支配收入和农民人均纯收入分别达到22180元和7640元左右，增长10%和12%。

——坚持以项目建设保增长、促转型，发展质量逐步提高。预计全年实施亿元以上项目740个，其中新开工450个、竣工180个。工业转型升级步伐加快。传统产业稳步提升，国机重工产业园、中信重工产业园等5个新园区项目，洛玻集团、白马集团、洛单公司等3个搬迁改造项目，500千伏洛阳西输变电工程等13个支撑项目开工建设；恩梯恩LYC精密汽车轴承产业园、南车集团城轨车辆组装、万基控股60万吨铝板带箔等一批项目竣工投产；洛阳石化1800万吨炼油扩能改造等项目前期进展顺利。战略新兴产业培育初见成效，中航锂电公司锂离子动力电池、普莱柯公司生物医药、恒生科技园等项目加快推进。预计信息产业销售收入比上年增长30%以上。国际文化旅游名城建设稳步推进。隋唐城国家遗址公园明堂项目建成开放，天堂项目主体封顶。龙门湿地公园一期等重点项目启动建设。牡丹文化节、河洛文化旅游节等圆满成功。全国旅游标准化试点城市、国家智慧旅游试点城市建设正式启动。我市入选福布斯中国（不含港澳台）最发达旅游城市。预计全年接待游客7765万人次，旅游总收入402.7亿元，分别比上年增长13%和15.7%。现代服务业蓬勃发展。信息服务业快速集聚，与河南联通公司共建的智慧洛阳项目正式启动，中移动呼叫中心、炎黄科技园等项目加快推进，动漫之都产业园等项目开工建设。现代物流业提速发展，新春都冷链物流中心、一拖物流中心等一批项目如期竣工，宏进农副产品国际物流中心、格力电器物流配送中心等一批项目加快推进。金融服务业迅速发展，新成立各类金融机构6家；省五大国有银行与我市战略合作累计履约资金1064.9亿元；通过召开银企洽谈会、创新融资方式等，全市新增融资450亿元。创新型试点城市建设加快推进。新建国家级研发机构3个、省级35个、市级51个，组建电子商务及互联网等产业技术创新联盟5个。实施重大科技项目195项，荣获国家科技进步奖7项、省科技进步奖22项。专利申请量比上年增长30%以上。我市荣膺国家知识产权示范城市称号。国家军民结合新型工业化产业示范基地通过评审。台湾微光机电（洛阳）基地等项目入驻科技城。新增省级创新型试点企业13家、高新技术企业15家，预计高新技术企业增加值比上年增长35.8%。产业集聚度不断提高。省批准11家产业集聚区新增规划面积48平方公里。经济开发区升格为国家级经济技术开发区，洛龙科技园升格为省级高新技术产业开发区。偃师、洛新产业集聚区入选省第二批新型工业化示范基地。全市17家产业集聚区新开工千万元以上基础设施项目200个，基本实现发展区基础设施全覆盖。完成固定资产投资920亿元，占全市的40%；规模以上工业主营业务收入3000亿元左右，占全市的56%。

——坚持以创建活动促提升、树形象，城市环境更加宜居。围绕创建全国文明城市、国家环保模范城市，全面加强城市规划、建设和管理，城市布局更加优化，辐射带动能力进一步增强。城市功能不断完善。第四期城市总体规划顺利获批，新增城市规划面积150平方公里。中心城区实现控制性详规全覆盖。龙门大道、310国道市区段改造等18条道路竣工通车，伊滨区主干路网框架基本形成，市区新建、改造道路通车里程105.4公里。新区污水处理厂建成试运行，伊滨区水厂、第一热源厂具备供水供热能力。城市区新增供热面积202万平方米，改造供水管网 38.8 公里 、居民水表15935户，新建、改造电力台区212个、公厕18座、垃圾中转站9座。城市品位持续提升。新开工城区改造工程2320万平方米，竣工860万平方米。完成背街小巷提升工程21项，改造老旧小区102个。市区重要道路实现机械化清扫作业。户外广告设置逐步规范。数字化城市管理系统进入调试阶段。生态建设成效明显。全市植树造林51.8万亩，抚育、改造森林37.8万亩。城市区新增公园绿地16万平方米。引黄入洛、引洛济秦、“两河一渠”治理工程加紧实施。伊滨公园龙门古韵段等项目建成投用。我市在全国首个通过水土保持生态文明城市验收。生态矿山建设和资源利用列入国家试点。综合交通体系日趋完善。洛栾高速、郑卢高速洛阳段建成通车，所有县（市）通高速目标如期实现。洛伊快速通道伊川段、洛吉快速通道一期建成通车，半小时经济圈初步形成。“四环一快一滨河”工程加紧实施。改造县乡公路 214 公里 。洛阳机场每周航班达到88架次，年旅客吞吐量超过70万人次，是上年的2倍。县城建设再掀高潮。县城改造、新区建设开工1250万平方米，竣工451万平方米。新修市政道路 229 公里 。偃师、宜阳、伊川、孟津等一批县城新区初具规模。

——坚持以深化改革促创新、转机制，发展活力明显增强。企事业单位改革深入推进。列入改革台帐的14家重点企业、85家市属事业单位及各县（市、区）均完成年度改革任务。政府融资平台优化整合，城投集团实现融资106.7亿元，12亿元企业债券顺利获批。事业单位绩效工资制度全面推开。我市荣获全国文化体制改革工作先进地区称号。开放活力进一步增强。签订招商引资合同项目420个，投资总额2043.5亿元。实际利用外资20.6亿美元、市外境内资金465亿元，分别增长16.3%和6.4%。山特维克华中技术服务中心、青岛啤酒等9个国内外500强企业落户我市。中信重工、一拖股份、洛钼集团A股首发上市，轴研科技实现再融资，累计募集资金50亿元。我市获批国家摩托车及零部件外贸转型升级基地、省级钢制办公家具出口基地。非公有制经济不断壮大。预计非公有制企业实现营业收入5053亿元左右、上交税金180亿元左右，分别增长15%和13%。非公有制经济占生产总值的比重达到53%左右。

——坚持以结构调整促增收、强基础，农业农村工作全面发展。“三篇文章”成效显著。粮食总产48.1亿斤，连续十年丰收。新发展蔬菜5.5万亩、花卉苗木14.5万亩、核桃18万亩，龙头企业13家、农民专业合作社371家。新增土地流转面积119.9万亩。发展休闲园区109家，带动就业2.8万人。转移农村劳动力135.7万人。农业综合生产能力不断提升。新增有效灌溉面积3.5万亩、节水灌溉面积6万亩，治理水土流失170平方公里。完成水库除险加固56座。新型农村社区建设扎实推进。开工新型农村社区试点63个。建设完善社区服务中心100个。升级改造农村电网工程257项。硬化通组通户道路 2126 公里 。扶贫开发力度不断加大。搬迁扶贫3176户、13383人。完成了6万人的脱贫任务。解决了农村35.2万人的饮水安全问题。

——坚持以管理创新促和谐、惠民生，群众生活水平进一步提升。福民实事全面完成。全年各级财政用于民生的支出达225亿元，占公共财政支出的67%。就业创业工作成效显著。发放小额担保贷款19.1亿元，培育微型企业700家，新增经济实体4.9万户。开展职业培训35.2万人。新增城镇就业12.3万人。社会保障体系进一步完善。城乡居民社会养老保险试点实现全覆盖。失业保险实现市级统筹。在全国率先与所有直辖市签订异地就医结算协议；在全省率先实行自付费医疗费用补助政策。城镇职工、城镇居民基本医疗保险和大额补充医疗保险年度最高支付限额大幅提高。新型农村合作医疗参合率达99.5%。城乡低保、农村五保户集中供养标准进一步提高，3076名孤儿纳入最低生活保障，123所农村敬老院实现集中供暖。开工保障性住房25969套、竣工12350套，7965户低收入家庭入住新居。教育保障水平不断提高。新建、改扩建中小学63所、幼儿园223所，引进民办学校86所。市管学校大班额比例下降58.2%，进城务工人员子女入学实现应入尽入。中小学信息化“班班通”工程、城市区中小学取暖降温工程进展顺利。职教园区基础设施逐步完善，6所院校加紧建设。医疗服务体系更加健全。在全省率先实现县级公立医疗机构药品零差率销售全覆盖，先看病、后付费模式全面推行。优势医院新增床位1260张。完成了6个县医院、7个乡镇卫生院的建设任务。所有村级卫生室纳入一体化管理。文化事业全面发展。新建社区文化活动中心25个、农家书屋1009个、社区服务中心212个。市图书馆、文化馆、美术馆和乡镇文化站全部免费开放。组织文化惠民演出600余场、公益电影3.7万场。为200个社区和行政村安装了体育健身器材。社会管理创新取得新进展。110应急联动服务实现市、县（区）两级全覆盖，群众诉求办理满意率达95%。城市社区网格化管理水平进一步提高，安全生产和食品安全形势总体平稳，社会大局保持稳定。

——坚持以作风建设促转变、提效能，发展环境不断优化。主动接受人大及其常委会监督，积极支持政协参政议政，市人大代表议案、建议和政协委员提案办理满意率均达98%以上。出台地方性法规3部。“作风转变年”活动深入开展，行政审批时限平均压缩32%。积极开展公开承诺和重点部门、重点科室、基层站所评议活动，创新电视问政等形式，解决企业、群众反映突出问题601个。加大工程建设等领域专项治理力度，公共资源交易中心挂牌运营。积极推进政务公开，定期在媒体公布市领导分包重大项目进展情况。双拥工作再创佳绩，军政军民关系更加融洽。反腐倡廉工作取得新进展。人口计生、民族宗教、对台、外事侨务、统计、审计、人防、气象、地震、档案、史志、残疾人工作、妇女儿童事业等都取得了新的成绩。

各位代表！一年来，洛阳发展的每一步足迹、每一个成就，都得益于省委、省政府和市委的正确领导，得益于市人大、政协和社会各界的监督支持，得益于全市人民的辛勤努力。一年来，我们在磨砺中经受了考验，在奋进中坚定了信心，在实干中凝聚了力量。在此，我代表市政府，向全市各条战线上的工人、农民、干部、知识分子和广大建设者，向人大代表、政协委员，向各民主党派、工商联、无党派人士和人民团体，向离退休老领导、老同志，向驻洛部队、武警官兵、公安干警，向所有关心支持洛阳发展的社会各界人士，表示崇高的敬意和衷心的感谢！

总结成绩的同时，我们也清醒地看到，由于对国内外经济形势的研判不够准确、对金融危机带来的冲击估计不够充分，年初确定的地区生产总值、外贸出口增速等目标未能完成。与此同时，经济社会发展中的矛盾和问题仍然较多：一是工业结构不尽合理。资源类、投资类产品比重过高，受金融危机的冲击较大，有色金属、硅光伏等支柱产业增速大幅下滑，企业效益明显下降。二是农业农村基础依然薄弱。农业科技、质量安全、市场流通等公共服务体系仍不健全，产业化水平较低，农民增收难度较大。三是城市精细化管理水平不高。城市功能尚不完善，交通拥堵问题有加重趋势，城市环境和文明程度有待进一步提升。四是体制机制障碍仍未得到彻底破解。部分领域改革改制不到位，遗留问题较多，需进一步加大攻坚力度。五是民生热点问题尚未得到妥善解决。就业、就学、就医、住房、社会保障等领域仍存在一些群众反映强烈的问题。六是机关作风与人民群众的期盼还有较大差距。一些干部的知识结构、领导方式和工作方法与新形势、新任务的要求不相适应，一些职能部门及工作人员服务意识不强，工作落实不力，行政效率低下，等等。这些问题，我们将采取更加有力的措施认真加以解决。

二、2013年总体要求及工作重点

今年是全面贯彻落实党的十八大精神、实施“十二五”规划的关键一年。世界经济持续低迷，国内经济发展中的不确定因素仍然很多。同时，我们更要看到，党的十八大胜利召开，中原经济区规划正式批复，国家继续实施积极的财政政策和稳健的货币政策，都为我市下一步的发展带来了难得的机遇，提供了持续增长的动力。我们一定要把思想和行动统一到党中央、国务院对国内外形势的分析判断上来，统一到省委、省政府和市委的决策部署上来，坚定福民强市目标不动摇，加快建设效益洛阳、创新洛阳、宜居洛阳、人文洛阳、和谐洛阳！

今年政府工作的总体要求是：全面落实党的十八大和中央、省委、市委经济工作会议精神，以提高经济增长质量和效益为中心，突出主题主线，抢抓机遇，稳中求进，持续提升，实干兴洛，全力打好“六加一”攻坚战，加快推进工业化、信息化、新型城镇化和农业现代化，进一步深化改革开放，强化创新驱动，加强生态建设，倾力改善民生，促进社会和谐，推动经济社会持续健康较快发展。

初步确定今年全市经济社会发展的预期目标是：地区生产总值增长11%左右，公共财政预算收入增长12%以上，固定资产投资增长23%左右，社会消费品零售总额增长16%左右，外贸出口增长10%左右，城镇居民人均可支配收入和农民人均纯收入均增长10%左右，城镇登记失业率控制在4%以内，居民消费价格涨幅控制在3.5%左右，人口自然增长率控制在6.5‰以内。

（一）着力推进产业协调发展，筑牢产业之基。以新型工业化为引领，大力发展服务业，加快发展现代农业，努力构建现代产业体系。

推动工业转型升级。加强与央企、省企的合作，着力引进、实施一批对调整结构、转型升级起支撑作用的重点项目，做到每个产业领域都有一批项目在建、一批项目投产，加快新兴产业培育与传统产业升级步伐，推动老工业基地振兴转型。一是优势产业要裂变扩张。年内实施亿元以上工业结构调整项目389个。在汽车及零部件、装备制造、食品、轻工、建材等领域，重点抓好南车集团城轨车辆二期、双瑞特种装备产业园、正大食品产业园等225个重大战略重组和新产品开发项目；在有色、化工、能源、纺织等领域，重点抓好洛阳石化1800万吨炼油扩能改造、华中铝业15万吨高档精密铝材等80个转型升级项目。加快实施中豪光电25万套摩托车照明系统等40个扩能项目。加快洛阳动力谷先进制造业示范基地建设，抓好中信重工产业园、轴研科技产业园等项目，确保麦达斯5万吨轨道车辆铝型材及车体大部件、北易公司20万套驱动电机和控制器等项目上半年建成。确保220千伏西工输变电工程等重点支撑项目年内建成投用。制订配套扶持政策，加强硅光电产品在政府机关、企事业单位的应用，帮助光伏产业渡过难关。二是战略新兴产业要加快培育。围绕新材料、新能源、电子信息、生物医药、智能装备与机器人、节能环保等领域，积极推动77个亿元以上重大项目建设。重点抓好微光机电技术芯片设计及制造等十大示范项目。力争天誉公司脱硫脱氮设备、613所光电产业园等一批项目开工建设，新兆电子导电玻璃、海斯迪公司聚合物锂电池等一批项目竣工投产。全年实现高新技术企业总产值1200亿元。三是产业集聚区建设要提质转型。提升产业集聚区配套服务功能，力争区内165个村庄全部启动迁并改造。加快高新区新型合金材料产业示范园区、洛龙光伏产业示范园区建设。争取新增2个省级战略新兴产业示范园区。实施集群培育工程，开工亿元以上主导产业项目150个，形成13个营业收入超百亿元的产业集聚区，确保主导产业、高新技术企业占产业集聚区营业收入的比重分别达到75%和35%以上。

促进服务业提质增速。金融、商务、物流、信息、会展等新兴服务业，是我市经济转型的重要载体、城市功能提升的有力支撑。要积极引进金融机构及后台服务中心在洛设立分支机构，年内实现村镇银行县域全覆盖。稳妥发展小额贷款公司，整顿规范担保公司，不断完善金融服务体系。结合旧城和城中村改造，优化涧西、西工、老城等传统商圈规划布局，推进一批美食、购物、休闲、演艺等特色商业街建设，实施一批高端综合体项目。大力发展咨询调查、职业中介、企业管理等现代商务，加快五洲国际工业博览城等商务项目建设，培育一批税收超亿元的特色商务楼宇。改造提升7个大型批发交易市场和农贸市场，促进大型连锁商贸企业向农村延伸。依托区域性综合交通枢纽，优化物流空间布局，提升物流业态，实施好国龙物流园区等一批重点项目。大力推进智慧洛阳建设，以电子政务建设为抓手，积极推进无线城市应用和开发，确保城区 3G 网络连续覆盖，初步实现热点区域 4G 网络覆盖。推动商贸流通企业电子商务应用，扩大网上销售份额。构建软件技术、动漫渲染等公共服务平台。抓好中移动呼叫中心等重点项目。加快信息通讯产业园等12个重点园区建设，吸引信息服务企业在洛建设总部或区域中心。在打造牡丹文化节等四季节会的基础上，积极引进国内外知名展会、赛会、年会落户洛阳，发展具有本地特色的品牌展会。同时，积极发展科技服务业、社区服务业，引导房地产业健康发展。

支持现代农业加快发展。落实强农惠农政策，加大涉农投入，持续做好“三篇文章”，着力提升农业规模化、基地化、市场化水平，促进农业稳定发展、农民持续增收。确保粮食总产稳定在40亿斤以上。大力实施种植业结构调整、畜牧业提升、农业产业化集群培育、农业龙头企业壮大、农业支撑体系建设五大工程，加快农业转型升级。新发展花卉苗木8万亩、蔬菜5万亩、水果2万亩、核桃6万亩。新（改）建标准化规模养殖场（区）70家。新培育市级以上龙头企业20家、农民专业合作社100家，农业产业化集群3－5个，休闲农业园区20家，争创一批国家级、省级名牌产品。加快农业科技集成应用，扩大基层农业技术推广体系覆盖面。新增农机动力5万千瓦，推广新型农机具1000台，尽快实现主要农作物生产全程机械化。完成30座中小型水库除险加固任务。治理水土流失150平方公里。

（二）着力传承特色化的城市文脉，彰显名城之魂。以打造河洛文化传承创新品牌、提高文化软实力为目标，深入挖掘历史文化内涵，加快建设国际文化旅游名城。

大力培育文化产业。围绕文化遗产活化创新，促进文化与旅游业深度融合。以龙门石窟世界文化遗产园区、隋唐城国家遗址公园等12个重点名城项目为龙头，加快实施东汉太学区域综合开发、河洛古城、动漫之都产业园等项目，着力打造一批文化产业集群。支持洛阳豫剧院、君兰动漫、报业集团等企业发展壮大，引进、培育一批有核心竞争力的知名文化企业。积极培育提升牡丹画、牡丹瓷、洛阳三彩等传统艺术和工艺美术产业。

大力提升旅游业发展水平。积极创建全国旅游标准化试点城市、国家智慧旅游试点城市，大力推进交通标识、星级饭店等九大重点行业提升工作，确保牡丹文化节前达到国家旅游标准化城市验收标准。健全旅游信息咨询等五大体系，实施旅游集散中心等五大示范工程，完善旅游基础设施，抓好西霞院等一批高端旅游产业集聚区建设，打造中原经济区旅游集散中心、国内外有较大影响力的旅游目的地。确保全年游客人数达到8600万人次，旅游综合收入485亿元。

大力完善公共文化服务体系。深入开展全国公共文化服务体系示范区创建工作。高标准筹建中国农耕博物馆、大运河博物馆。积极推进大运河和丝绸之路申报世界文化遗产工作。抓好市科技馆、曲剧院等项目建设。加快市群艺馆、方志馆、职工文化活动中心等项目的前期工作，争取早日开工。抓紧实施县（市）图书馆、文化馆、社区文化活动室等一批文化基础设施项目。加强文化人才队伍建设，大力扶持文艺精品创作生产。组织好公益电影放映、百场公益演出等文化惠民活动。

（三）着力构建现代化的城镇体系，加快城乡一体化进程。按照“一中心三板块五组团四支撑”的总体布局，全面提高城镇化质量，努力构建功能完善、内涵丰富的新型城镇化体系。

提升新区辐射带动力。千方百计拓宽融资渠道，加快重大市政基础设施和公益性项目配套建设，确保牡丹文化节前希望大道、洛宜铁路改线、开元大道东段景观水系等一批项目顺利竣工，新区商务功能区中心水系出形象。加快龙门大道定鼎门遗址区域等重要节点建设。行政副中心要尽快完善规划，高起点建设，彰显特色，提升品质，打造亮点。

增强洛北城区综合承载力。加快完善中心成网、外围成环、立体交叉、四周放射的城市路网体系。确保牡丹文化节前唐宫西路、丽春东路等10条道路建成通车，6月底前滨河路全线贯通、九都快速路开工建设。做好启明南路跨洛河大桥项目前期工作。综合提升25条背街小巷和120个老旧小区。加大城市电力、自来水、燃气管网改造和公交场站、消防队站等基础设施建设力度。加快城市热力工程建设，尽快解决老城区、瀍河区等区域供热率过低问题。按照“依法、阳光、和谐、安全”的要求，稳步推进旧城和城中村、城郊村改造，加快建设安置房。

提高城市精细化管理水平。坚持建设与管理并重，全面推进数字化管理和社区网格化管理，健全城市管理长效机制。持续开展门店招牌、户外广告、临街建筑立面、占道经营、车辆乱停乱放、建筑垃圾清运等专项整治活动。市区主次干道全面实现机械化清扫。实施城市畅通工程，完善智能交通系统，加快停车场、人防过街通道建设，新增停车位1万个，下大力气缓解城市交通拥堵，让群众出行更加便捷、舒适。

完善区域性综合交通体系。全年实施20个总投资324亿元的交通基础设施项目。加快连霍高速洛阳至三门峡段改扩建、新310国道、洛吉快速通道二期、洛伊快速通道市区段、机场改扩建等工程建设。确保牡丹文化节前东环路一期、西环路涧西段主线竣工通车，6月底前南环路开工建设。实施19条省道提升改造项目。继续优化航线结构，争取开通1条国际航线，做好赴台包机直航试点工作，提升航空吞吐能力。

统筹城乡一体化发展。完善市县融合发展机制，加快县城和中心镇建设，壮大县域经济实力，完善教育、医疗、文化、商贸等公共服务设施，增强承载和辐射带动能力。逐步解决农民工在城镇就业、居住、社保、子女入学等方面遇到的问题。加快推进基础设施、公共服务向农村延伸，改善农民群众生活质量。稳步推进新型农村社区试点建设。新（改）建县乡公路 200 公里 ，硬化通组通户道路 2000 公里 。解决25万农村人口的饮水安全问题。确保30%的行政村完成环境集中整治任务。加快汝阳等片区扶贫开发。实施整村推进50个，搬迁扶贫3000户、12500人。

（四）着力建设生态化的城市环境，打造宜居之都。牢固树立集约、绿色、低碳发展理念，构建人、城、山水和谐共处的生态城市，让洛阳的天更蓝、水更清、环境更优美。

积极创建国家环保模范城市。以PM2.5监测为契机，完善环境监测体系，全面治理市区工业污染、燃煤锅炉、机动车尾气、建筑工地等重点污染源，确保全年城市区空气质量优良天数达到310天以上。完成大唐洛热等4家企业脱硝工程。凡治理无望、主要污染物或重金属长期不能稳定达标排放的企业，坚决予以关、停、转、迁。加强固体废弃物的处置管理。搞好秸秆转化利用，确保综合利用率达到95%以上。加快污水处理厂建设，力争伊滨区污水处理厂上半年开工，涧西、瀍东污水处理厂升级改造工程11月底前竣工投用。建成栾川、新安、汝阳县城第二污水处理厂和产业集聚区污水处理厂。加强城市河渠两岸截污工程建设，尽快完善安乐、杨文、新街等片区管网设施，两年内实现城市区污水管网全覆盖，消除城区污水直排河渠现象。严厉打击各类环境违法行为。

积极建设低碳城市。突出重点领域，倡导全民节能，严格项目节能评估和审查。完成11家重点企业节能改造，淘汰、关闭39家落后小企业。抓好锅炉窑炉改造、电机系统节能等技改项目，积极推进建筑供热计量及节能改造。在城市公交、市政、出租车行业逐步推行使用新能源汽车。开展循环经济、资源综合利用，实施洛阳循环经济园区和新安、宜阳等产业集聚区循环化改造项目。积极申报“城市矿产”示范基地。力争全年单位生产总值能耗下降3%。

积极提升林业生态市建设水平。巩固扩大天然林保护、退耕还林成果，完成造林49.3万亩，抚育、改造森林27.1万亩。大力实施环城防护林、生态廊道、城郊森林公园、公共绿地和道路绿化提升工程，加快伊滨公园建设，提升洛浦公园品位，确保城市中心区绿化覆盖率达到36.5%，人均公园绿地面积达到 8.4 平方米 。加快引黄入洛、引洛济秦工程进度，两年内“四河七渠”全部通水。

（五）着力推进改革创新，建设对外开放新高地。进一步深化改革、扩大开放，加快国家创新型试点城市建设，促进各种创新要素、生产要素加速集聚、合理流动。

加大改革攻坚力度。深化企事业单位改革改制。加快推进事业单位养老保险制度改革。选择符合条件的公益性单位，探索建立法人治理结构。稳妥推进厂办大集体改革。健全城市供水、供气、公交等市政公用行业特许经营制度及相关行业市场准入、监管制度。深化县域医药卫生、食品药品监管等体制改革。创新基本公共服务提供方式，扩大政府购买服务范围。深化投融资体制改革，做大做强政府性融资平台。完善土地保障机制，优先支持重大项目、重点企业和保障性住房等建设用地。

加快推进科技创新。大力实施中小高新技术企业成长、创新型产业集群构建等八大科技创新工程，以科技创新引领产业结构调整。围绕产业集聚区主导产业、龙头企业亟需的关键技术，深入开展产学研合作。重点实施基因工程新型动物疫苗等100个技术创新项目、六轴工业机器人等100个产业化项目。整合产业链科技资源，加快大学科技园、科技城等重点园区建设，谋划建立战略新兴产业技术研究院、机器人和创意设计等产业技术创新联盟，支持北航科技园等专业孵化器和企业加速器建设，提高科技成果转化率。强化企业创新主体地位，提升企业创新能力，支持企业与科研单位、高等院校共建研发中心和合作项目。新建省级以上企业技术中心、工程实验室10家以上，市级企业技术中心10家，新增创新型（试点）企业3家，专利申请量突破6500件。持续加强质量提升和品牌带动工程建设。

进一步扩大对外开放和合作。紧盯世界500强、国内500强企业的产业布局及动向，寻找机遇开展战略合作。围绕装备制造、铝、钛、轴承、锂离子电池、生物制药等产业的延链和补链，强力实施重点产业链招商攻坚计划。围绕战略新兴产业、文化旅游、物流、商贸、现代农业等领域，积极开展专业化、集群式招商。促进外贸转型升级，力争在电子信息、装备制造、太阳能光伏、新材料、铝深加工、钢制办公家具等优势产业，引进、培育一批出口导向型项目，扶持一批重点企业扩大进出口规模。积极推进洛阳综合保税区、省级纺织品出口基地的申报工作。

不断提升非公有制经济发展水平。全面落实支持非公有制经济发展的政策措施，继续实施“小巨人”企业培育工程。完善中小企业公共服务平台，缓解企业融资难、销售难、引进人才难等问题。鼓励支持民间资本投入重大基础设施、科技研发、战略新兴产业、社会事业、民生改善等领域。推动100个亿元以上非公有制经济项目建设，确保非公有制经济增加值增长15%以上。

（六）着力实施福民惠民工程，构建幸福之城。认真办好福民实事，切实解决好与群众生活密切相关的就业、入学、就医、社保等问题，让洛阳人民生活得更有保障、更有尊严、更加幸福。

千方百计扩大就业。认真落实各项创业扶持政策，完善创业服务体系，扩大小额担保贷款基金规模，促进全民创业，确保新增城镇就业10万人以上。进一步提高社会保障水平。加快全民健康信息保障工程建设。开展新农合补充医疗保险试点，逐步将特殊慢性病等纳入保险范围，提高参合农民保障水平。加强城乡低保、社会救助工作，确保困难群众及时得到救助。完善养老服务体系，加快19个社会办养老服务项目建设，新增养老床位2600张，为全市147所农村敬老院和5所公办社会福利机构全部安装供暖设施。扎实推进利用住房公积金支持保障性住房建设试点城市工作，开工保障性住房3.2万套，建成1.2万套。坚持优先发展教育。继续实施学前教育和普及高中教育三年行动计划。新建、改扩建中小学校26所、幼儿园155所，确保学前一年幼儿入园率达到85%、小学适龄人口入学率达到100%、初中适龄人口入学率达到99.8%。严格落实义务教育学校划片招生、就近入学等制度，缓解“大班额”等突出问题。加大对农村、特别是深山区中小学教育的扶持力度。加强学校法制和道德教育。逐步理顺城市区义务教育阶段学校管理体制。整合职业教育资源，引导高等院校、职业学校调整专业设置，加快培养经济转型和社会发展所需的紧缺人才。支持河南科技大学建设一级学科博士点，实现地 方高校 博士点零突破。推进基本公共卫生服务均等化。加快县（区）医院建设。优势医院新增床位2000张。完成135个乡镇卫生院取暖工程、17个乡镇卫生院达标改造和10个县级卫生监督中心建设。加强平安洛阳建设。完善信访稳定风险评估、矛盾纠纷排查调解机制，积极化解信访积案，提升110联动服务水平，切实解决好群众合理诉求。深入推进社会管理创新，强化整体联动功能。严厉打击各类违法犯罪，增强人民群众的安全感。强化企业安全生产主体责任，深化隐患排查治理，夯实基层基础工作，确保安全生产不出大的问题。抓好食品药品安全工作，让群众吃得放心、吃得健康。同时，深入开展双拥共建工作，积极支持驻洛部队、武警部队、民兵预备役和人防建设，促进军民融合发展。办好市第十二届运动会，开展全民健身活动。提高出生人口素质，促进人口均衡发展。积极支持工会、共青团、妇联等人民团体工作。持续抓好民族宗教、对台、外事侨务、统计、审计、气象、地震、档案、史志、残疾人事业等工作。

三、政府自身建设

各位代表！四年前，大家在这里共同审议通过了本届政府的工作目标。职责所系，不容丝毫懈怠；重任在肩，更当百倍努力。我们一定要忠实履行向全市人民作出的庄严承诺，以更加饱满的激情、更加务实的作风，勤勉尽责，开拓进取，全面建设人民满意的服务型政府，圆满完成本届政府的目标任务，向全市人民交上一份满意的答卷！

坚持依法行政。牢固树立依法治市理念，提高各级干部运用法治思维和法治方式深化改革、推动发展、化解矛盾、维护稳定的能力。强化城乡建设、安全生产、社会治安、食品药品安全等领域的行政执法，切实做到严格、规范、公正、文明。自觉接受市人大及其常委会的法律监督、工作监督和市政协的民主监督，主动接受社会公众、新闻媒体监督。加强和完善政府立法，拓展公民有序参与立法的途径，提高立法质量和水平。优质高效办理人大代表议案、建议和政协委员提案，认真听取和采纳各民主党派、工商联和各人民团体、无党派人士的意见。完善决策咨询、社会听证、重大事项评估等制度，依法规范行政行为，让每一项政府决策、每一个行政行为，都凝结着民主的智慧、闪耀着法治的光芒！

坚持放权简政。深化行政审批制度改革，清理和缩减行政审批收费项目，将行政审批权限逐步向县（区）下放，确保该简化的程序优化到位，该减免的费用兑现到位，该下放的权限落实到位，真正给基层松绑、为企业减负、还群众方便。严肃查处损害发展环境的违法行为，坚决纠正不正之风，让洛阳真正成为投资者的沃土、创业者的乐园。

坚持务实勤政。以“狠抓落实年”活动为契机，将中央“八项规定”落实到政府工作的方方面面。时刻惦记群众的安危冷暖，多到困难和矛盾集中、群众意见多的地方去，多办群众看得见、摸得着的实事好事。深入开展争创“人民满意的公务员集体”、“人民满意的公务员”活动，努力打造一支作风硬、素质高、能力强的公务员队伍。大力精简会议、文件，转变会风、文风，提倡开短会、讲短话、行短文。切实减少不必要的接待应酬，把更多的精力放在调查研究、解决问题上，把有限的财力用在发展经济、改善民生上。坚决不讲空话、套话，坚决不上形象工程！

坚持高效施政。加强行政服务中心建设，优化再造工作流程，完善并联审批机制。积极推行网上办公、网上审批和行政监察，提升政务服务水平。健全绩效考评和行政问责制度，加大对重点工作、重要事项、重大项目、民生工程及群众诉求的督导落实力度，确保政令畅通，确保每一项政府决策都落到实处。

坚持廉洁从政。深入开展各类专项治理活动，认真解决好损害群众利益的突出问题。积极推进政务公开、“三公”经费公开，让权力在阳光下运行。坚持厉行节约，严格控制评比、达标、表彰活动和公费出国、公务购车等经费支出。充分发挥公共资源交易中心的作用，进一步规范工程建设招投标、经营性土地使用权出让、产权交易、政府采购等工作。健全政府系统惩治和预防腐败体系，严厉查处各类违法违纪案件，让廉洁从政成为每一位公职人员的行为自觉。

各位代表！今年是本届政府任期的最后一年。前进的道路只有起点，没有终点。让我们在十八大精神的指引下，在省委、省政府和市委的正确领导下，开拓创新，团结奋进，务实重干，为建设幸福美丽新洛阳、全面走在中原经济区建设前列而努力奋斗！

祝大家新年快乐、身体健康、工作顺利、阖家幸福！
